# Correlation of Maternal Thyroid Stimulating Hormone Levels With Lipid Profile in Pregnant Women With Hypothyroidism

**DOI:** 10.7759/cureus.37748

**Published:** 2023-04-18

**Authors:** Alka Yadav, Ranjan Katyal, Shilpa Mittal, Tapan Kumar Saha

**Affiliations:** 1 Biochemistry, Al-Falah School of Medical Science and Research Centre, Faridabad, IND; 2 Biochemistry, Noida International Institute of Medical Sciences, Noida, IND; 3 Biochemistry, Amrita School of Medicine, Faridabad, IND

**Keywords:** triglycerides, total cholesterol, second and third trimester, pregnancy, hypothyroid, lipid profile, thyroid hormone, hdl-c, ldl-c, tsh

## Abstract

Introduction

Pregnancy leads to changes in hormonal levels and lipid profile. Thyroid hormones play a crucial role in embryonic growth and fetal development. Untreated thyroid disease during pregnancy can lead to a high risk of complications.

Aim

The aim of the study is to examine the correlation between thyroid stimulating hormone (TSH) and lipid profile in pregnant women with hypothyroidism.

Materials and methods

This cross-sectional case-control study was conducted at the Biochemistry Department, Alfalah School of Medical Science & Research Centre, Dhauj, Faridabad, Haryana, India. The study consisted of 500 patients (250 cases and 250 controls) who fulfilled the inclusion and exclusion criteria. Of the 250 cases recruited, 23 cases were in the 2nd trimester and 209 cases were in the 3rd trimester. Blood samples were collected from the participants to assess their lipid profile and TSH levels.

Results

The study showed a statistically significant difference between the mean TSH levels of hypothyroid pregnant females in the 2nd trimester (3.85 ± 0.59) and the 3rd trimester (4.71 ± 0.54). There was a significant positive correlation observed between TSH and Total Cholesterol, Triglycerides, and LDL-C in both the 2nd and 3rd trimesters. In the second trimester, there was a significant positive correlation observed between TSH & TC (r = 0.6634, p<0.0005), TSH & TG (r= 0.7346, p=0.00006), TSH & LDL (r= 0.5322, p= 0.008). In the third trimester, there was a significant positive correlation observed between TSH & TC (r = 0.8929, p<0.00001), TSH & TG (r= 0.430, p<0.00001), TSH & LDL (r= 0.168, p= 0.015). However, no significant correlation was found between TSH levels and HDL-C in either trimester. The correlation coefficient and p-value for TSH & HDL were r = 0.2083, p=0.340 in the second trimester, and r = 0.0189, p=0.2384 in the third trimester.

Conclusion

A significant increase in TSH levels in hypothyroid pregnant women was observed in the 3rd trimester compared to the second trimester. Moreover, a significant positive correlation was found between TSH and lipid profile (total cholesterol, triglycerides, and LDL) in both trimesters, but not with HDL. These findings highlight the importance of monitoring thyroid hormone levels in the later stages of pregnancy to avoid potential maternal & fetal complications.

## Introduction

Pregnancy demands an increased supply of metabolic fuel, leading to changes in hormonal levels and alterations in the lipid profile of pregnant women [1]. Thyroid hormones play a crucial role in embryonic growth and fetal development [2,3] It is well known that thyroid function undergoes significant transformations throughout pregnancy [4-6].

From 12 to 14 weeks of gestation, the fetus utilizes maternal thyroid hormones. After that, the fetus's thyroid gland begins functioning on its own. In pregnant women, hypothyroidism can have negative effects on both the mother's health and the health of the fetus. A lack of thyroid hormones can negatively impact a child's mental development, potentially resulting in mental retardation and cognitive delays in early childhood [7].

Untreated thyroid disease during pregnancy can lead to a high risk of complications, including an increased likelihood of miscarriage, placental abruption, hypertensive disorders, and growth retardation. It is important to note that thyroid disease is the second most common endocrine disorder among reproductive-age women [8].

During a normal pregnancy, there are expected changes in lipid metabolism and an increase in lipid concentration as the pregnancy progresses [9]. However, these changes are not typically considered to have an adverse effect on cardiovascular health as they quickly drop back to pre-pregnancy levels after delivery [10,11].

Determining the difference between physiological and pathological lipid changes during pregnancy can be complex. Mild elevations in the lipid profile are observed in the early months of pregnancy, while significant elevations are seen in the third trimester [12]. Both triglycerides (TG) and total cholesterol (TC) are transported to the placenta, where they are metabolized and then sent to the developing fetus [13].

The understanding of the role of thyroid stimulating hormone (TSH) in normal pregnancy is crucial for evaluating the impact of abnormal TSH function on both obstetric outcomes and fetal health. The effect of TSH on lipid profile in hypothyroid pregnant women remains unclear in the Indian population. This study aims to investigate the relationship between TSH and lipid profile in pregnant women with hypothyroidism.

## Materials and methods

Study design

This cross-sectional case-control study was conducted in the Department of Biochemistry at AFSMS & RC, Dhauj, Faridabad.

Patient selection

The patients were selected according to the inclusion and exclusion criteria. The study included pregnant women who visited the Obstetrics OPD for the first time as participants while excluding those with a history of diabetes mellitus, metabolic disorders, lipid disorders, thyroid disorders, oncological disorders, or who were taking hormone replacement therapy or thyroid medications.

Methods

Venous blood samples were obtained from each subject after an overnight fast of 14-16 hours. The sample was then processed for TSH levels and lipid profile. The TSH levels were measured using an automated VIDAS instrument while the lipid profile was estimated using an automated Erba Transasia automated analyzer and commercial kits. TSH value was used to diagnose hypothyroidism state in accordance with American Thyroid Association (ATA) recommendations [14]. The National Cholesterol Education Program-Adult Treatment Panel III Guidelines were used to classify the serum lipid levels of the participants in the study. According to these guidelines, hypercholesterolemia was defined as having a total cholesterol (TC) value greater than 200 mg/dl, high low-density lipoprotein (LDL) as having a value greater than 100 mg/dl, hypertriglyceridemia as having a triglyceride (TG) value greater than 150 mg/dl, and low high-density lipoprotein (HDL) as having a value less than 40 mg/dl. The presence of one or more than one abnormal serum lipid value was defined as dyslipidemia.

Study population

This study comprised a total of 500 pregnant women. A total of 250 pregnant women diagnosed with hypothyroidism were included in the case group and 250 age and sex-matched healthy pregnant women were recruited in the control group. The gestational age of the women was calculated using two methods: the first day of their last normal menstrual period and ultrasonography. The first, second, and third trimesters of pregnancy were defined as the periods of up to 14 weeks, 15-28 weeks, and 29-40 weeks of gestational age, respectively.

Statistical analysis

The collected data was summarized and compiled into a master chart using MS Excel 2007. The quantitative data were expressed as mean ± SD. Qualitative data were expressed as a percentage of the total sample size. Quantitative data of the test and control group were compared by unpaired student’s t-test. The chi-square test was used to examine the relationship between the qualitative variables. Pearson’s correlation coefficient was used to find a correlation between variables. A p-value < 0.05 was considered statistically significant.

Ethical considerations

The study was conducted after obtaining approval (AFSMS&RC/F-01/20/104) from the Ethical Committee of AFSMS & RC, Dhauj, Faridabad.

## Results

Table -1 presents the mean and standard deviation values of various lipid profile parameters and thyroid-stimulating hormone (TSH) for the second and third trimesters of pregnancy. The statistical significance of the difference between the mean values of the two trimesters was evaluated using a p-value.

**Table 1:** Mean distribution of biochemical parameters in the 2nd and 3rd trimesters among hypothyroid pregnant females. Values are expressed as Mean and SD. p < 0.05 - significant, p < 0.01 - Highly significant, p ≥ 0.05 - non-significant, TSH: Thyroid stimulating hormone

Parameters	Mean ± SD [2^nd^ Trimester]	Mean ± SD [3^rd^ Trimester]	p-value
Total Cholesterol (mg/dL)	352.78 ± 35.57	379.04 ± 15.97	p < 0.0001
Triglyceride (mg/dL)	456.13 ± 60.14	508.23 ± 21.05	p < 0.0001
HDL Cholesterol (mg/dL)	39.91 ± 4.79	39.51 ± 4.87	p = 0.7084
LDL Cholesterol (mg/dL)	163.91± 42.71	191.54 ± 38.63	p < 0.0001
VLDL Cholesterol (mg/dL)	91.22 ± 12.02	101.65 ± 4.20	p < 0.0001
TSH (µIU/mL)	3.85 ± 0.59	4.71 ± 0.54	p < 0.0001

The results show that there was a significant increase in the levels of total cholesterol, triglycerides, LDL cholesterol, and VLDL cholesterol in the third trimester compared to the second trimester. However, there was no significant difference in the levels of HDL cholesterol between the two trimesters. Moreover, the TSH levels were significantly higher in the third trimester compared to the second trimester.

Table 2 shows the relationship between the thyroid-stimulating hormone (TSH) and the lipid profile in the second trimester of pregnancy for women with hypothyroidism. The table displays the correlation coefficient (r-value) and the p-value for each lipid parameter, including total cholesterol, triglycerides, HDL, and LDL. A positive correlation was observed between TSH and total cholesterol, triglycerides, and LDL. No significant correlation was found between TSH and HDL. The p-values indicate the level of significance of these correlations with lower p-values indicating a higher level of significance.

**Table 2:** Correlation of serum TSH with lipid profile among the 2nd trimester hypothyroid pregnant females p < 0.05 - significant, p < 0.01 - Highly significant, p ≥ 0.05 - non-significant TSH: Thyroid stimulating hormone

Parameters	Total Cholesterol	Triglyceride	HDL	LDL	VLDL
Serum TSH	r = 0.6634	r = 0.7346	r = 0.2083	r = 0.5322	r = 0.7346
	p = 0.0005	p = 0.00006	p = 0.340	p = 0.008	p = 0.00006

Table 3 shows the relationship between TSH and lipid levels in pregnant women who have hypothyroidism during their third trimester of pregnancy. The results indicated a statistically significant positive correlation between TSH and total cholesterol, TG, and LDL cholesterol. There was no significant relationship found between TSH levels and HDL cholesterol levels.

**Table 3:** Correlation of serum TSH with lipid profile among the 3rd trimester hypothyroid pregnant females p < 0.05 - significant, p < 0.01 - Highly significant, p ≥ 0.05 - non-significant TSH: Thyroid stimulating hormone

Parameters	Total Cholesterol	Triglyceride	HDL	LDL	VLDL
Serum TSH	r = 0.8929	r = 0.430	r = 0.0189	r = 0.168	r = 0.4309
	p < 0.00001	p < 0.00001	p = 0.2384	p = 0.015	p < 0.0001

## Discussion

Hypothyroidism is a common condition during pregnancy, and it has been associated with several adverse maternal and fetal outcomes. One of the potential complications of hypothyroidism during pregnancy is dyslipidemia, which is characterized by an abnormal lipid profile [14].

Despite a growing body of research on the relationship between TSH levels and lipid profile in hypothyroid pregnant women, several studies have reported different results. While some studies have found a positive correlation between TSH levels and total cholesterol, LDL cholesterol, and triglycerides, others have found no significant association or even an inverse relationship. These discrepancies in the literature could be due to differences in study design, sample size, population characteristics, and the methods used to measure thyroid function and lipid profile

Our study aimed to investigate the correlation between TSH and lipid profile in hypothyroid pregnant women. We recruited a total of 500 pregnant women for this study. Two hundred fifty pregnant women diagnosed with hypothyroidism were included in the case group and 250 age and sex-matched healthy pregnant women were taken as the control group. We measured and correlated the serum TSH levels with the lipid profile, including total cholesterol, LDL cholesterol, HDL cholesterol, and triglycerides for the 250 recruited cases.

Gupta et al (2021) investigated the association between hypothyroidism during pregnancy and dyslipidemia. They found that pregnant women with hypothyroidism had significantly higher levels of total cholesterol, LDL cholesterol, and triglycerides compared to pregnant women without hypothyroidism. The study also found that TSH levels were significantly correlated with total cholesterol, LDL cholesterol, and triglyceride levels [15].

Mahantesh et al (2020) found that TSH levels are positively correlated with total cholesterol and suggested regular estimation of TSH and lipids during pregnancy to minimize maternal-fetal complications [16].

In our study, we found a positive correlation between serum TSH levels and total cholesterol, triglycerides, LDL cholesterol, and VLDL in hypothyroid pregnant women in the second & third trimesters of pregnancy. However, we didn’t find any significant correlation between serum TSH levels and HDL cholesterol in hypothyroid pregnant women in the second & third trimesters of pregnancy.

To the best of our knowledge, this study is the first to investigate the link between lipid profile and thyroid-stimulating hormone in pregnant women with hypothyroidism residing in a rural region of northern India. Additionally, this study assessed both TSH & lipid profiles simultaneously. However, there are certain limitations to consider, the findings of our study are solely based on data obtained from a single institution. Therefore, further studies are necessary to confirm our results before generalizing them to the wider population of pregnant women with hypothyroidism. Also, the timing of measurements may be important, as changes in thyroid function and lipid metabolism may occur throughout pregnancy. Moreover, since our study's design was a case-control one, we can only establish a correlation between maternal TSH and lipid profile in pregnant women with hypothyroidism, and not causation.

## Conclusions

In our study, we observed a significant elevation in TSH levels among pregnant women during the third trimester when compared to those in the second trimester. However, we did not detect any significant changes in the levels of HDL cholesterol between the second and third trimesters in these women. A significant positive correlation was found between TSH & total cholesterol, and triglycerides & LDL-C in both trimesters. In conclusion, our study contributes to the mounting evidence supporting the association between TSH levels and the lipid profile in pregnant women with hypothyroidism. We explored the relationship between TSH levels and lipid profile in pregnant women with hypothyroidism, and we took potential confounding factors like age into account as it can impact lipid metabolism and thyroid function. These findings underscore the importance of screening for hypothyroidism during pregnancy and monitoring lipid levels to minimize the risk of maternal and fetal complications. Consequently, a larger prospective study is imperative to validate our findings and explore the potential benefits and risks of using thyroxine replacement therapy as a targeted intervention for dyslipidemia in hypothyroid pregnant women.
